# The Use of Functional Biomaterials in Aesthetic and Functional Restoration in Orbital Surgery

**DOI:** 10.3390/jfb15020033

**Published:** 2024-01-29

**Authors:** Kevin Y. Wu, Jamie K. Fujioka, Patrick Daigle, Simon D. Tran

**Affiliations:** 1 Department of Surgery, Division of Ophthalmology, University of Sherbrooke, Sherbrook, QC J1G 2E8, Canada; yang.wu@usherbrooke.ca (K.Y.W.);; 2 Faculty of Medicine, Queen’s University, Kingston, ON K7L 3N6, Canada; 3 Faculty of Dental Medicine and Oral Health Sciences, McGill University, Montreal, QC H3A 1G1, Canada

**Keywords:** functional biomaterials, biomedical devices, physicochemical properties of biomaterials, ophthalmology, orbital floor fracture, orbital implants and prostheses, material science, patient outcomes

## Abstract

The integration of functional biomaterials in oculoplastic and orbital surgery is a pivotal area where material science and clinical practice converge. This review, encompassing primary research from 2015 to 2023, delves into the use of biomaterials in two key areas: the reconstruction of orbital floor fractures and the development of implants and prostheses for anophthalmic sockets post-eye removal. The discussion begins with an analysis of orbital floor injuries, including their pathophysiology and treatment modalities. It is noted that titanium mesh remains the gold standard for orbital floor repair due to its effectiveness. The review then examines the array of materials used for orbital implants and prostheses, highlighting the dependence on surgeon preference and experience, as there are currently no definitive guidelines. While recent innovations in biomaterials show promise, the review underscores the need for more clinical data before these new materials can be widely adopted in clinical settings. The review advocates for an interdisciplinary approach in orbital surgery, emphasizing patient-centered care and the potential of biomaterials to significantly enhance patient outcomes.

## 1. Introduction

The utilization of functional biomaterials within the realms of oculoplastic and orbital surgery marks a significant convergence of material science and clinical innovation. In this review article, we present a synthesis of primary studies published from 2015 to 2023. This comprehensive literature review is designed to provide a detailed exposition of the applications of functional biomaterials in the repair of orbital floor injuries and the crafting of orbital implants and prostheses post-eye removal procedures.

This review begins with an overview of orbital floor injuries, covering their pathophysiology, incidence, and impact, then shifts to treatment indications and the scenarios where advanced materials prove most effective. It evaluates the ideal characteristics of biomaterials, emphasizing the need for a balance between mechanical strength and biological compatibility. The current standards and alternatives for orbital floor repair are critically examined. The article also explores the use of biomaterials in creating orbital implants and prostheses for anophthalmic sockets post-eye removal, assessing their integration and functionality.

The novelty of this review lies in its unique clinical insights and the establishment of a link between material science and clinical practice. While other reviews have addressed aspects of orbital floor repair and the use of orbital implants and prostheses, they often lack a clinical perspective that considers the translational pathway from bench to bedside. Our article incorporates input from clinicians, thereby bridging this gap and offering a perspective that encompasses the entire spectrum—from the foundational science to practical application and beyond. It elucidates the barriers to clinical translation and discusses future directions, potentially guiding the next wave of advancements in orbital surgery.

## 2. Use of Functional Biomaterials in the Repair of Orbital Floor Injuries

### 2.1. Overview of Orbital Floor Injuries and Reconstruction

Among the most frequent emergency consultations related to trauma are cases of orbital trauma, often connected with injuries to the facial bones and soft tissue, leading to orbital floor fractures (i.e., blowout fractures) [1]. Orbital floor fractures typically result from impacts involving objects larger than the orbital aperture. The hydraulic and buckling mechanisms, theories suggesting rapid intraorbital pressure increase or deformation of the inferior rim, respectively, explain how these impacts cause fractures, potentially leading to the prolapse and entrapment of orbital contents into the maxillary sinus [2,3,4,5]. Patients might exhibit a range of symptoms including eyelid ecchymosis, edema, pain and restrictions in eye movement, vertical diplopia, and, in particular, discomfort in the inferior orbit during upgaze, which may indicate the entrapment of the inferior rectus muscle [1]. Additional signs can include enophthalmos and ptosis, usually associated with large fractures where orbital soft tissues descend into the maxillary sinus. Other notable symptoms include infraorbital nerve distribution hypoesthesia and orbital and eyelid emphysema [1,6].

### 2.2. Indications for Treatment

While the majority of orbital floor fractures can be managed through observation, allowing for the natural resolution of edema and orbital hemorrhage, some cases necessitate surgical intervention. Surgical indications generally include persistent diplopia with limitations in upgaze and/or downgaze within 30° of the primary position and positive forced duction tests [1,6,7]. These symptoms collectively suggest functional entrapment of tissues impacting the inferior rectus muscle. Other indicators for surgery are enophthalmos exceeding 2 mm, considered aesthetically unacceptable, and large fractures involving at least half of the orbital floor [1,6,7].

The preferred surgical approach often involves an inferior transconjunctival incision. This method encompasses the elevation of the periorbita from the orbital floor, the liberation of herniated extraocular muscles and fats from the fracture, and the placement of an implant over the fracture to prevent recurrent herniation into the fractured site [8,9,10].

The selection of implant materials for this procedure can range from alloplastic—including porous polyethylene, nylon foil, polytetrafluoroethylene, silicone sheet, or titanium mesh—to autogenous options like split cranial bone, iliac crest bone, or fascia. The versatility of alloplastic implants, especially when combined with synthetic and metallic components, facilitates microplating and provides an effective strategy for managing large fractures encompassing the orbital floor or the medial wall. On the other hand, autogenous grafts, while beneficial, require an additional operation site, which often leads to their less frequent utilization [7,8].

Though the optimal material for orbital reconstruction remains a subject of debate among ophthalmic and maxillofacial surgeons, significant advancements in biomaterials for orbital floor repair have been made in recent years, a development that we will delve into in the ensuing section [8].

### 2.3. Ideal Properties of Biomaterials for Orbital Floor Repair

The objective of orbital wall reconstruction is the restoration of normal anatomical relations within the internal orbit. To achieve this, a wide range of materials have been utilized. Yet, the search for an ideal biomaterial continues, guided by specific desirable attributes:

- Biocompatibility and safety: The material should be non-allergenic and non-carcinogenic. It should mimic the physical properties of the tissue it is replacing.
- Long-term acceptance: The material should be permanently accepted by the body.
- Chemical stability: It should be chemically inert, and capable of being sterilized without deteriorating its chemical properties. The choice of biomaterial should consider its inherent antibacterial properties.
- Manipulability and stability: The material should be easily manipulated during surgical procedures and retain its form post-implantation.
- Fixation capability: The material should allow for secure fixation to the host bone using screws, wire, suture, or adhesive.
- Non-potentiating: It should not encourage microbial growth or the resorption of the underlying bone. It should also not distort adjacent structures.
- Radio-opacity: For clear post-surgical evaluation, the material should be radiopaque.
- Cost-effectiveness: Particularly for alloplastic materials, the cost should be reasonable.
- Porosity: This characteristic is vital for promoting tissue ingrowth and vascularization. Higher porosity enhances cell infiltration, nutrient exchange, and integration with surrounding tissues.
- Mechanical strength and elasticity: These properties should match those of native tissue to prevent implant failure or tissue damage.
- Appropriate biodegradability: Biodegradable materials should enable gradual replacement by native tissues at a rate that aligns with tissue healing and remodeling processes.
- Optimal surface roughness: The surface roughness of certain materials, such as titanium, has the potential to enhance bone-to-material contact, resulting in accelerated osteointegration and increased adhesion strength. However, it is crucial to strike a balance, as heightened surface roughness also poses a risk of bacterial adhesion to the materials.
- Hydrophilicity: Hydrophilic biomaterials offer advantages such as enhanced water retention, promoting cell adhesion and tissue integration, but careful consideration is necessary to avoid excessive hydrophilicity, which could increase susceptibility to biofilm formation.

### 2.4. Current State: Current Gold Standard, Alternative Options, and Types of Biomaterials in Orbital Floor Repair

An overview of the key features, advantages, and disadvantages of current biomaterials used for orbital floor repair is described below and summarized in Table 1.

#### 2.4.1. Autogenous Bone

Autogenous bone involves sourcing tissue from various areas, such as the mandibular coronoid process, anterior maxillary wall, mandibular symphysis, rib, scapula, cranium, and iliac crest. Bone tissue offers strength, flexibility, and radio-opacity but can vary in resorption and modeling capability, challenging orbital fracture repair. Other notable drawbacks include complex shaping, donor site morbidity, and unpredictable resorption. In 2019, Saha and colleagues conducted a study comparing outcomes of orbital floor reconstruction for blowout fractures using either autologous bone grafts from the iliac crest or mandible, or alloplastic implants like silastic blocks or titanium mesh. The study involved 30 patients, with early correction rates for diplopia and enophthalmos at 71.42% for the iliac crest graft and 100% for other methods. At the 12-week mark post-operation, all groups exhibited complete correction. Notably, complication rates were 20% for mandible grafts and 12.5% for silastic blocks, while titanium mesh and iliac grafts had no complications [11]. Recently, Moubarak and collaborators compared autologous cranial bone grafts to titanium mesh for explosive orbital fractures, revealing that while autologous grafts have limitations such as constrained donor sites and potential complications, they remain immunologically favorable [12]. Despite the reported drawbacks, autologous bone grafts continue to be favored for their biocompatibility, moldability, and low immune reactivity, although their outcomes can be influenced by surgical experience.

#### 2.4.2. Autogenous Cartilage

**Figure 1:**
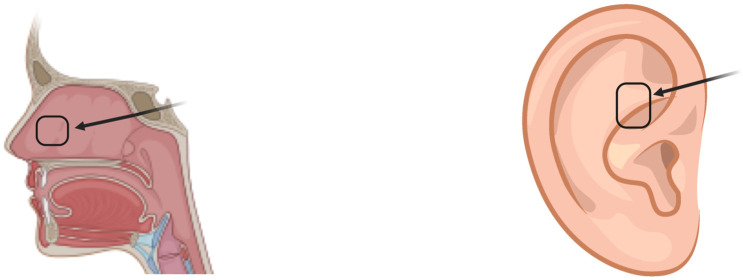
This figure would depict donor sites for autogenous cartilage, including the auricular cartilage and nasal septum.

Autologous cartilage stands out as a viable option for orbital floor reconstruction due to its accessibility, flexibility, and documented non-resorption [13]. Particularly suitable for smaller defects, it is sourced from areas, such as costal cartilage, auricular cartilage, and nasal septum (Figure 1). This material offers easy harvesting, contouring, lasting support, minimal resorption, and reduced donor site morbidity [14,15,16,17]. The nasal septum and conchal cartilage emerge as primary sources for orbital reconstruction [15,16]. Mangan and colleagues conducted a retrospective study on 25 patients who underwent orbital fracture repair using either nasoseptal cartilage grafts or titanium mesh implants. Both groups showed comparable long-term outcomes, with resolved diplopia and improved ocular motility [14]. Similarly, Deep and team assessed the use of auricular conchal cartilage for repairing orbital floor fractures with minimal bone loss, demonstrating significant improvements in eye movement, positioning, and diplopia, suggesting its efficacy for functional and aesthetic restoration [15]. However, cartilage autografts share drawbacks with autologous bone, including lacking radio-opacity and offering less structural support. Challenges also arise from cartilage’s inherent memory and tendency to revert to previous shapes, making it difficult to maintain new shapes within the orbit [13,18].

#### 2.4.3. Allogenic Materials

Allogenic materials, which encompass allografts, homografts, and xenografts, offer osteoinductive and/or osteoconductive properties despite lacking living cells. These materials integrate into host tissue, providing a scaffold for tissue growth. Notably, they bring advantages over autologous grafts, such as lack of donor site morbidity, reduced surgery time, pre-fabrication potential, and ample supply. Studies on lyophilized dura have proven its ability to avoid infections or extrusion, but enophthalmos rates of 5.4–20% have been reported [19,20]. Demineralized allogenic bone grafts have shown no graft-related complications, inflammation, or infections [21]. However, allogenic grafts have higher resorption rates compared to autologous grafts and pose the risk of disease transmission, hindering their broader clinical application [19,20,21,22,23,24,25].

#### 2.4.4. Alloplastic Materials

A range of alloplastic materials, including titanium, ceramics (such as bioactive ceramic glass, calcium phosphate, hydroxyapatite, tricalcium β phosphate, aluminum oxide, and calcium sulfate), and plastics (like acrylates and porous PE), are used for craniofacial fracture reconstruction. These diverse alloplastic materials have gained popularity for orbit reconstruction due to their user-friendliness and reduced surgical complications. They eliminate the need for donor sites, shorten operative time, and are readily accessible. However, their downsides include being foreign bodies that can induce host reactions, potentially necessitating implant removal in the case of rejection. Oliver and team found that out of 11 studies involving 585 patients, a total of 25 surgical complications occurred, representing a 4.3% complication rate. These complications included infection, inflammation, graft migration or explantation, and hematoma. Specifically, porous PE implants had the highest infection rate at 2.0%, while Poly-L-lactic acid (PLLA) implants had the highest rate of graft explantation at 5.9%. Overall, explantation was necessary for 0.6% of all implants (three cases) [26].

#### 2.4.5. Metals

Metallic materials, with titanium being the most widely used, remain the preferred gold standard for orbital floor reconstruction nowadays [27,28,29,30]. Titanium’s exceptional biocompatibility, corrosion resistance, and mechanical properties closely resembling natural bone make it a preferred choice. Titanium mesh, available in various thicknesses, is especially advantageous for extensive defects in orbitozygomatic or orbitofrontal reconstructions, as custom-shaped implants can be effectively leveraged using titanium micro-screws through a retroseptal transconjunctival approach [31]. Advanced techniques using cone-beam computed tomography (CT) can aid in precise positioning, reducing radiation compared to standard CT [30,32].

Several studies have documented that titanium mesh offers comparable or superior outcomes to autogenous materials [12,14]. However, manual adaptation during surgery can be time-consuming and error-prone, especially for less experienced surgeons. Associated complications include implant rupture, corrosion, screw weakening, and bone resorption [33]. In addition, fibrotic adherence between titanium implants and orbital structures, although rare, can lead to diplopia and eyelid retraction [27,30]. Shaping and bending titanium mesh plates can also be challenging. Techniques like direct metal laser sintering offer avenues to address these challenges, paving the way for continued advancements of this material in orbital fracture management [34].

The recent literature underscores the innovation in prebending titanium mesh to enhance its application in orbital fracture repair, particularly through the utilization of stereolithographic models. This method leverages the mirroring of an unaffected orbit’s anatomy over the damaged one to generate a preformed mesh. Transitioning from these conceptual frameworks, contemporary research has delved into the practicalities of personalized orbital implants, predominantly focusing on the role of 3D models for implant shaping and direct 3D-printed implants [35,36,37,38,39,40]. Chai et al. (2021) used 3D-printed models of patient fractures as templates in intraoperative implant trimming [41], demonstrating a significant reduction in surgical time and complications. Similarly, digitally reconstructing the affected orbit and superimposing it on the implant allows it to act as an outline around which the surgeon can precisely cut [42,43]. In particular, autologous bone grafts are fragile and difficult to manipulate. Vehmeijer et al. (2016) addressed this by using 3D templates, which they found enhanced accuracy and efficiency while also being cost-effective [43].

Studies have also explored the utilization of 3D-printed models as molds. Contrary to templates where the 3D model serves as an outline, molds allow the implant to be directly worked into the 3D model to provide an accurate form. This type of pre-operative bending has widely been researched using titanium mesh [44,45,46,47,48] and polyethylene plates [49]. These studies commonly conclude that this procedure is beneficial and enhances outcomes when compared to traditional methods.

#### 2.4.6. Hydroxyapatite (HA)

Hydroxyapatite (HA), a calcium phosphate salt analogous to bone mineral, is a widely used biomaterial in craniofacial reconstruction. HA demonstrates excellent biocompatibility, mechanical bonding with host bone, and limited resorption [50,51,52]. For orbital reconstruction, porous biphasic beta-tricalcium phosphate (b-TCP)/HA plates have shown high biocompatibility and stability in animal models due to their fibrous tissue in-growth capacity, making them a viable alternative to autologous grafts [53]. However, its low tensile strength and brittleness make it less suitable as a bone substitute. The difficulty in stabilizing HA implants and their relative incompatibility with rigid fixation limit their use for primary orbital fracture treatment. Further, a study of 405 patients by Nam and colleagues found that post-operative enophthalmos was significantly more frequent in HA-treated patients compared with porous PE [54]. Similarly, Mathur and collaborators encountered intraoperative failures and post-operative infections among 35 patients with 46 sites of reconstruction using HA or carbonated apatite [50]. Thus, while HA holds promise in orbital floor reconstruction, its tensile strength, brittleness, and complication risks may make this material less promising than other alternatives.

#### 2.4.7. Non-Absorbable Polymers

##### Silicone

Silicone, a widely used material for almost 50 years, is valued for its inertness, flexibility, ease of handling, and cost-effectiveness. In 2010, Prowse and colleagues retrospectively assessed 81 patients who had orbital floor reconstruction, comparing silicone implants (58 patients) with non-silicone materials (autografts, titanium mesh, and resorbable plates) (23 patients). Silicone implants showed benefits like reduced palpability, fewer post-operative complications, and a decreased need for subsequent surgeries, leading to positive outcomes attributed to silicone’s inertness and capsule formation [55]. However, over the years other studies have revealed significant implant-related complications such as cyst formation, infection, extrusion, and displacement [56,57,58]. Consequently, silicone has fallen out of favor for orbital floor repair.

##### Polytetrafluoroethylene (PTFE)

Polytetrafluoroethylene (PTFE), known for its biological inertness and moldability, has also been reported as a suitable material for orbital reconstruction. Studies involving ePTFE (Gore-Tex) and FEP-ePTFE (reinforced with fluorinated ethylene propylene) in animal models have demonstrated their effectiveness in treating smaller orbital fractures. Their biocompatibility and efficacy have also been shown to be comparable to HA and autogenous bone grafts [59,60]. Zhou and colleagues conducted a retrospective analysis of 32 patients who underwent hand-carved ePTFE intraorbital reconstruction, which demonstrated significant improvements in enophthalmos. Their approach proved safe and effective, suggesting ePTFE is a viable alternative for late post-traumatic enophthalmos [61]. However, scattered case reports have highlighted late complications with these implants, such as complex fistula formations, prompting a shift towards newer materials [62].

##### Porous Polyethylene (PE)

Porous ultra-high-density PE (Medpor^®^) has been successfully used to repair orbital defects over the past two decades. PE’s suitability for orbital floor reconstruction is attributed to its durability, biocompatibility, insolubility, minimal tissue reactions, and high tensile strength. Commercially available PE sheets of varying sizes and thicknesses can be easily adapted for individual cases, promoting tissue ingrowth while minimizing complications such as foreign body reactions [63,64,65]. A recent comparative study of Medpor versus titanium mesh by Marella and colleagues found that Medpor significantly lowered pain and enophthalmos scores, while both groups had comparable outcomes for all other parameters [28]. While it lacks radiodensity and might not be easily visualized on post-operative CT scans, Medpor is generally well-received by the body and its porous architecture enables the formation of fibrovascular networks, serving as a safeguard against infections and implant displacement [66]. Other research has confirmed that its clinical outcomes are generally favorable, although some cases of immediate and long-term complications, such as surgical site infection, cyst formation, delayed atraumatic hematoma, and implant extrusion, have been reported [26,67,68,69,70].

The introduction of patient-specific 3D-printed models (3DP) has enhanced the application of PE by streamlining surgical procedures and reducing the risk of implant failure. Before 3DP models were available, estimating the size and shape of orbital floor blowout fracture defects relied on 2D CT scans, and visualizing the posterior landing zone during surgery through small trans-conjunctival incisions was challenging. This often led to implant retrieval for further shaping and placement adjustments, potentially causing implant fatigue and prolonged operative times. In two cases described by Pang and collaborators, 3DP orbital floor bone models facilitated the precise shaping of porous PE implants, allowing direct visualization and accurate defect matching during transconjunctival incisions. This technique significantly reduced operative time, anesthesia duration, and the risk of implant failure [71].

##### Polyethylene/Titanium Composite

The Medpor Titan implant, a combination of titanium mesh and porous PE, leverages titanium’s strength, radio-opacity, and memory along with PE’s ability to integrate with fibrous tissue. Notably, its innovative design features a smooth surface on both sides, eliminating the need to grind or smooth sharp titanium edges to prevent abrasions. Research highlights outcomes using this biomaterial for orbital fractures, where Peng and colleagues retrospective analysis found similar efficacy and complication rates between titanium-only and PE/titanium hybrid implants [72]. Similarly, Tabrizi and colleagues demonstrated that the Medpor Titan implant effectively reinforced and stabilized the orbital region while restoring orbital volume [73], while Blessing and team have shown this material improved visual acuity, improved diplopia, and reduced enophthalmos [74]. Another retrospective study by Lee assessed the timing of surgical repair with the Medpor implant, showing improved outcomes and fewer complications when the procedure was performed within 2 weeks post-injury [63]. Collectively, these studies indicate that the innovative Medpor Titan implant demonstrates promise in addressing orbital fractures by offering enhanced stability, orbital volume restoration, and favorable surgical outcomes. However, other research has reported instances of late-onset infection, suggesting the need for caution when using this biomaterial [67].

##### Polyethylene Reinforced with Hydroxyapatite (HAPEX™)

High-density PE reinforced by HA (HAPEX™) has been recognized for its stiffness, osteoconductivity, and biological inertness, although its brittleness is a noteworthy drawback. Downes and colleagues applied HAPEX in orbital floor cases and demonstrated the biomaterial’s capacity to stimulate bone integration with the implant. HAPEX materials displayed excellent stability upon palpation and a complete absence of extrusion. Furthermore, CT images revealed seamless integration between the implant and the supporting bone, leaving no room for gaps. However, the deficiency of adequate strength, modulus, and toughness in HAPEX imposes limitations on its usage as a load-bearing bone substitute [75].

##### Smooth Nylon Foil

Smooth nylon foil (SupraFOIL), a relatively recent addition to orbital surgery, has been proven to be safe and effective for orbital floor reconstruction with a low complication rate [76,77]. This non-absorbable clear sheeting is derived from standard nylon suture biomaterial. In a retrospective study by Park and colleagues, involving 181 patients who underwent orbital fracture repair using nylon foil, their results demonstrated that these implants effectively repaired fractures. The study employed a transconjunctival approach and single-screw fixation, revealing that implant fixation could potentially reduce the incidence of hemorrhage within the implant capsule for non-porous implants [77]. More recently, Campbell and colleagues conducted a retrospective review of 80 patients who underwent isolated orbital floor fracture repair with the use of a wraparound nylon foil implant. They observed no instances of significant post-operative enophthalmos in small and medium-sized fracture groups and only one case of this in the large fracture group. There were also no instances of implant-related complications. The authors expressed a preference for using nylon over other materials, such as titanium, to prevent orbital fixation syndrome, a condition characterized by restricted eye movement due to scarring or adhesions within the orbit [76]. Despite its promising use, other studies have reported complications with nylon, such as orbital hematoma and local dense fibroconnective tissue with associated pyogenic granuloma and lymphoplasmacytic inflammation [78,79].

#### 2.4.8. Absorbable Polymers

##### Polycaprolactone

A recent study by Kim showcased 3D-printed biodegradable PCL implants, confirming their safety, functionality, and aesthetic suitability for orbital fracture repair. The study found that PCL mesh’s characteristics are well-suited for orbital wall reconstruction, offering both structural stability through its semi-rigidity and precise anatomical adaptation due to its malleability, which surpasses that of other biodegradable implants. Notably, caproic acid, a PCL monomer, was found to hydrolyze into metabolites with mild acidity (pH 5), making it safer compared to metabolites of other implants [36].

Jung and Kim conducted a study comparing uHA/PLLA and PCL meshes for orbital fracture repair, focusing on their distinct handling properties. Among the 30 patients in each group, eye movement remained unrestricted 6 months after surgery. The PCL group exhibited no instances of diplopia or enophthalmos, whereas the uHA/PLLA group had two cases of diplopia and one instance of enophthalmos. Anatomical accuracy was high in both groups, and remarkably, no significant differences in surgical outcomes or complications were found [80].

Furthermore, a periosteum and PCL complex, where the periosteum is applied to a HA-poly (l-lactide–ɛ-caprolactone) sheet, has shown promising clinical results, providing a potential alternative to autologous bone for orbital reconstruction. A pilot study led by Asamura and colleagues compared this biomaterial to autologous ilium-based biomaterials for orbital floor defect reconstruction. Both groups exhibited normal eyeball position and movement post-surgery, leading the authors to consider the PCL composite as a promising autologous bone substitute [81].

##### Polylactic Acid-Based Materials

Polylactic acid (PLA), a high molecular weight bioresorbable osteosynthetic material, has demonstrated its utility in orbital fracture management since the 1990s [82]. This biomaterial has the benefit of both alloplastic and autologous implants, such as ease of contouring in thermolabile forms, mechanical integrity while the polymer resorbs, and avoidance of donor-site morbidity [83]. PLA offers unique benefits, leaving a stable shelf of healed bone or soft tissue after complete resorption, unlike permanent implants [84]. Initially, polymerized poly(L-lactide) (PLLA) systems were employed due to their crystallinity and hydrophobicity rendering them resistant to hydrolysis, preventing full bioresorption and loss of strength within the initial two years of implantation. Early animal and human studies have demonstrated that PLLA’s use within the orbit can lead to positive healing outcomes without significant inflammation [85,86]. Comparative research by Al-Shukan and Lindquist showed no significant differences between autologous bone and PLA for fractures exceeding 2 cm^2^ [83]. Long-term studies have also confirmed this biomaterial does not cause abnormal tissue reactions on CT [84]. In 2021, Esmail and colleagues conducted a prospective case series involving 22 patients with orbital floor blow-out fractures to evaluate the use of PLA (Resorb X) for repair. They found that these implants resulted in improved elevation, diplopia, and enophthalmos in most cases, although 36.4% had late enophthalmos after initial improvement. Radiological improvement in orbital vertical height was observed in all cases in the first month, but significant late floor bowing occurred in 45.5% of cases after 1 year, and implant resorption was noted in 45.5% of cases. They concluded that, while PLA provided good outcomes for small defects, there was significant floor bowing in medium-sized defects after 1 year, indicating potential limitations in their use [87].

Another combination biomaterial of unsintered HA/poly-L-lactide (u-HA/PLLA), was investigated for intermediate-term safety and effectiveness in orbital fracture reconstruction. A retrospective analysis of 240 patients who underwent orbital fracture repair using various biomaterials revealed that silicone sheets had more post-operative complications compared to u-HA/PLLA sheets. Deformities were significantly higher in silicone sheets (28.6%) and PLLA/poly-glycolic acid mesh (31.9%) than in u-HA/PLLA sheets. Clinical observations showed new bone formation and the incorporation of u-HA/PLLA in remodeled bone. This biomaterial’s absorption was also not found to delay healing or cause significant complications. They concluded that u-HA/PLLA and titanium were superior to non-absorbable options in terms of safety and effectiveness [88].

##### Polyglycolic Acid-Based Biomaterials

Research indicates that a biodegradable copolymer of PLA and polyglycolic acid (PGA) (Lactosorb) degrades at a faster rate (9–15 months) compared with PLLA and therefore may be better suited for orbital repair [89]. While PGA’s rapid degradation within 2 months and substantial resorption (>90%) within 9 months makes it less suitable for standalone use, PLA/PGA composites exhibit more optimal resorption rates and enhanced long-term structural support. Hollier and collaborators employed resorbable PLA/PGA mesh plates to reconstruct orbital defects larger than 1 cm^2^ in 12 patients, with 9 patients followed up for an average of 6 months. Enophthalmos occurred in two cases shortly after surgery due to technical errors in mesh placement, and an inflammatory reaction necessitated implant removal in one patient after 7 months. Their study suggests that PLA/PGA mesh is suitable for specific orbital floor reconstructions, cautioning against its use for larger defects or over the infraorbital rim due to potential risks [90]. Another investigation by Lin and colleagues demonstrated successful outcomes in 29 cases of common orbital floor fractures repaired using PLA/PGA plates, with 6 patients encountering complications like transient diplopia and persistent enophthalmos. Overall, the study suggests that resorbable PLA/PGA plates are generally effective and safe for orbital floor fracture repair, except in severe orbital trauma cases where sturdier implants might be more appropriate [91].

Polyglactin 910, commonly known as Vicryl, is a resorbable synthetic material composed of PLA/PGA in a 1:9 ratio that has been commonly used as a material for surgical sutures. Both film and mesh forms of polyglactin 910 have been used for orbital fracture repair with mesh being preferred due to its layered structure making it easy to customize [92,93]. Mauriello and colleagues reported on its use for orbital floor fractures in 28 patients over 5 years, achieving varied thickness (6–56 layers) and size through folding and cutting. While considered advantageous, its flimsiness was evident as 56 layers were needed for effective repair, and it was associated with low-grade inflammatory reactions lasting up to 11 months [93].

##### Polydiaxanone (PDO)

Plate, foil, and sheet forms of polydioxanone (PDO) have also been explored for orbital floor reconstruction, with mixed outcomes reported in clinical practice [94,95,96,97,98,99]. In a study involving 24 patients with small orbital defects, Becker and colleagues compared collagen and PDO, showing successful outcomes without complications. However, stability was an issue for larger defects, leading the investigators to conclude it is only suitable for smaller defects (<2 mm) [95]. Iizuka and colleagues reported positive outcomes with PDO plates for 1 to 2 cm defects with a connection to the maxillary sinus in 20 patients who tolerated the biomaterial well, though overcorrection sometimes led to transient diplopia [97]. Gierloff and colleagues studied PDO foil in the orbital floor repair of 194 patients with different defect sizes, proving its success with minimal issues in ocular movement, nerve sensitivity, and globe position, even for larger defects exceeding 2 cm^2^ [96]. Conversely, Bauman and team encountered complications such as hematoma, diplopia, extrusion, and enophthalmos when using this biomaterial [98]. In a prospective study by Kontio and colleagues, a PDO implant was used for reconstructing the internal orbital wall in 16 patients with various fractures. Their findings showed no muscle entrapment or bone growth in the orbital wall, but unsatisfactory shape, volume restoration, and scar formations in some cases [99]. Thus, the use of PDO for orbital reconstruction is controversial and inadvisable in some studies.

##### Polyglactin 910/PDO Copolymers

For several years, flexible membranes composed of polyglactin 910/PDO have been marketed globally under the trade name Ethisorb (Johnson & Johnson) [100,101,102,103,104]. In their investigation, Büchel and colleagues examined 87 patients, revealing that 21 individuals (24.1%) encountered post-operative complications. They also deduced that this biomaterial proves efficacious for mending minor-to-moderate orbital floor fractures, with a maximum size limit of 2 × 2 cm [100]. Conversely, a more recent study by Steinmassl and colleagues compared the application of polyglactin 910/PDO for small and large isolated orbital floor fractures in 61 patients, and found no significant differences in clinical outcomes based on defect size [101]. Similarly, research by Tabrizi and collaborators found that defect size did not have any effect on the stability of polyglactin 910/PDO plates used to repair orbital blow-out fractures. However, larger defects did lead to slower degradation [103]. A long-term follow-up study by Blake and colleagues found that using polyglactin 910/PDO in 70 patients with small-to-moderate fractures led to minimal diplopia (9%), periorbital swelling (6%), and no enophthalmos at suture removal with no persistent complications after 18 months post-operation [102]. Gerressen and colleagues examined the effectiveness of the Ethisorb patch and PDO foil for restoring orbital geometry in 21 patients with extensive comminuted and defect fractures. The surgical intervention using these materials did not lead to significant changes in orbital geometry, and a reduced diplopia rate was observed [104]. Thus, while earlier studies highlighted size limits, recent research demonstrates that defect size might not significantly impact clinical outcomes.

**Table 1:** Current Biomaterials for Orbital Floor Repair.

Material Type	Key Features	Advantages	Challenges	References
Autologous Materials				
Autologous bone	-Sourced from mandibular coronoid process, anterior maxillary wall, mandibular symphysis, rib, scapula, cranium, and iliac crest	-Intrinsic strength -Flexibility Radio-pacity -Biocompatibility -Tissue tolerance after implantation -Low immune reactivity	-Complex shaping -Donor site morbidity -Unpredictable resorption -Limitations on available donor sites	[11,12,13]
Autologous cartilage	-Primarily sourced from nasal septum and conchal cartilage for orbital floor repair but also harvested from auricular and rib cartilage	-Easier to harvest and contour compared to bone -Lasting support -Minimal resorption even after several years -Minimal donor site morbidity	-Lacking radio-opacity -Less structural support than bone -May revert to its previous shape	[13,14,15,16,17,18]
Allogenic Materials				
Titanium	-Wide use in orthopedics and craniofacial reconstruction -Cone-beamed CT can aid in precise positioning -Direct metal sintering techniques emerging to overcome challenges related to these materials	-Good biocompatibility -Corrosion resistance -Mechanical properties resembling bone -Suitable for permanent stability in large defects (i.e., orbitozyggomatic or orbitofrontal reconstructions) -Comparable or superior outcome to autogenous materials	-Requires manual adaptions during surgery which can be time-consuming or error-prone -Associated complications include implant rupture, corrosion, screw weakening, and bone resorption -Fibrotic adherence between titanium materials and orbital structures can lead to diplopia and eyelid retraction -Challenges with shaping and bending	[27,28,29,30,31,32,33,34]
Hydroxyapatite (HA)	-Calcium phosphate salt analogous to bone material -Widely used in craniofacial reconstruction	-Excellent biocompatability -Limited resorption	-Low tensile strength -Brittleness -Difficulty stabilizing HA implants -Incompatibility with rigid fixation -Associated with post-operative enophthalmos, intraoperative failures and infections	[50,51,53,54]
Non-Absorbable Polymers				
Silicone	-Used in orbital reconstruction for nearly 50 years	-Biologically and chemically inert -Flexible -Easy to handle -Cost-effective material -Positive post-operative outcomes such as reduced infection and need for repeat surgeries	-Risk of implant-related complications such as infraorbital cyst formation, infection, extrusion, and implant displacement	[55,56,57]
Polytetraflouroethylene (PTFE)	-Used in orbital reconstruction for smaller defects (<1.5 cm)	-Biologically and chemically inert -Non-antigenic -Sterilizable via autoclaving -Easily moldable -Proven safe and effective for post-traumatic enophthalmos	-Some reports of late complications such as fistula formations -Less evidence on reliability since it is not used as frequently	[59,60,61,62]
Polyethylene (PE; Medpor)	-Used in orbital floor repair over the past two decades -Porous structure that vascular components and connective tissue can grow into -Enhanced by patient-specific 3D printed models	-Customizable material -Porous structure enables formation of fibrovascular networks -Reduced infection and implant displacement risks	-Reports of immediate and long-term complications, such as surgical site infection, cyst formation, hematoma, and implant extrusion	[26,28,63,64,65,66,67,68,69,70,72,73]
Polyethylene/Titanium (Medpor Titan)	-Combination of titanium mesh and porous PE	-Leverages titanium’s strength, radio-opacity, and memory along with PE’s ability to enable fibrovascular ingrowth -Smooth surface reducing abrasions -Enhanced stability	-Reports of late-onset infection	[63,67,72,73,74]
Polyethylene with hydroxyapatite (HAPEX)	-High-density porous PE reinforced by HA	-Biologically inert -Can stimulate bone integration -Good stability -Good integration between implant and supporting bone	-Brittleness -Inadequate strength, modulus, and toughness to substitute load-bearing bone	[75]
Nylon foil (SupraFOIL)	-Non-absorbable clear sheeting derived from nylon suture biomaterial	-Found to be safe and effective -No findings of post-operative enophthalmos in small and medium-sized fractures -May prevent orbital fixation syndrome	-Some cases of intracapsular hemorrhage, orbital hematoma, and orbital inflammation reported	[76,77,78,79]
Absorbable Polymers				
Polycaprolactone (PCL)	-Semi-rigid mesh structure	-Structural stability -Highly malleable enabling precise anatomical adaptation -Hydrolyzes into metabolites with mild acidity	-Some complications observed	[36,103,104]
Polylactic acid (PLA) based materials	-High molecular weight -Bioresorbable osteosynthetic material	-Ease of contouring -Mechanical integrity -Avoidance of donor-site morbidity -Stable shelf life of healed bone or soft tissue after complete resorption -Resistant to hydrolysis -Comparable outcomes to autologous bone	-Limitations in use for medium or larger-sized defects	[57,82,83,84,85,86,87,89]
Poly glycolic acid (PGA) biomaterials	-Biodegradable polymer -Rapid degradation within 2 months and >90% resorption within 9 months making it less suitable for standalone use -Often used in combination with PLA	-Suitable for orbital floor repair of smaller defects and where faster degradation (within 6 months) is needed -Low risk of delayed infection or migration	-Not suitable for large defects -Less suitable for severe orbital trauma cases due to lack of stability	[89,90,91,92,93]
Polydiaxanone (PDO)	-Semicrystalline polymer -Available in plate, foil, and sheet forms	-Positive outcomes for smaller defects	-Evidence of post-operative complications such as hematoma, diplopia, extrusion, and enophthalmos -May be suboptimal for larger defects	[94,95,96,97,98,99]
Polyglactin 910/PDO (Ethisorb)	-Flexible membranes that offer strength and long-term resorption	-Positive results for small to moderate-sized orbital floor defects	-May be unsuitable for larger-sized defects	[100,101,102,103]

### 2.5. What’s New? Emerging Biomaterials and Their New Applications in Orbital Floor Repair

An overview of the key features, advantages, and disadvantages of emerging biomaterials used for orbital floor repair is described below and summarized in Table 2.

#### 2.5.1. Additives and Coatings

In the rapidly evolving field of reconstructive surgery, emerging biomaterials are revolutionizing the approach to orbital floor reconstruction, offering innovative solutions that combine structural support with biocompatibility to restore both form and function to this intricate anatomical region. Additive manufacturing techniques are increasingly employed for surface modification across biomaterials like metals, ceramics, and polymers, encompassing both physical and chemical methods. For instance, surface-treated titanium has been explored extensively in recent years, such as alkali treatment and thermo-chemical processes inducing HA formation. Porous coatings on titanium surfaces play a crucial role in promoting osseointegration. Notable methods for creating these coatings include microarc oxidation (MAO), dealloying, and 3D printing [105].

#### 2.5.2. Nanoparticles

The use of nanoparticles in the context of orbital floor repair represents another innovative approach to improving the outcomes of orbital reconstructions. Nano materials, characterized by their dimensions ranging from 1 to 100 nanometers, are tailored to exhibit high biocompatibility through surface chemistry and functionalization with specific biomolecules. The application of nano materials in orbital floor repair involves intricate bio-physicochemical interactions at the nano-bio interface. These interactions are influenced by various nanoparticle characteristics, such as the material’s chemical composition, surface functionalization, shape, porosity, surface crystallinity, and hydrophobicity or hydrophilicity. The repair and regeneration of the orbital floor bone, a natural composite material comprising organic collagen fibrils and inorganic hydroxy carbonated apatites, can be effectively achieved using synthetic HA [106]. The nanoscale morphology, porosity, and crystallinity of HA, determined by functional modifications, influence its chemical and biological properties.

Alhamoudi and his team utilized polyurethane (PU) and HA in varying sizes (both micro and nano) and proportions (25%, 40%, and 60%) to devise bioactive scaffolds aimed at orbital repair and regeneration. Their research explored cell viability, collagen production, VEGF levels, and scaffold vascularization. Their findings highlighted the effective integration of HA, emphasizing surface concentration, interconnected pores, and enhanced mechanical strength when 40% nano-HA was incorporated. These attributes, along with notable biocompatibility and vascularization, indicate its potential for effective orbital floor regeneration [106]. Interestingly, these outcomes align with prior research by Patel et al. who demonstrated that HA nanoparticles within cyclic acetal hydrogels promoted positive in vivo bone growth when used for orbital defect repairs [107]. In another study, Alhamoudi and collaborators developed HA synthesized with ionic substitutions (fluoride, carbonate, and citrate) with reduced particle size compared to standard commercial and stoichiometric hydroxyapatites. Their findings underscored the potential of incorporating fluoride and citrate ions into the HA lattice structure to boost bioactivity and cell viability, signifying a promising avenue for future orbital bone repair and regeneration research [108].

Incorporating antibacterial metal ions into porous structures is also an emerging strategy to enhance osseointegration. Specifically, nanoceria (cerium oxide nanoparticles) have been investigated for their strong antibacterial abilities, arising mainly from the production of H_2_O_2_ due to shifts between the Ce^3+^ and Ce^4+^ oxidation states. The higher Ce^3+^/Ce^4+^ fraction in cerium nanoparticles enhances the catalytic activity of the nanoparticles, effectively sustaining intracellular oxygen levels and consequently fostering angiogenic induction. Within this context, Sarfaraz and his team developed silk fibroin scaffolds enhanced with HA and Ce-doped ZnO nanoparticles through the freeze gelation technique. These scaffolds underwent comprehensive testing including FT-IR, micro-CT, and mechanical assessments to evaluate their porosity, swelling, degradation, and antibacterial properties. Notably, in vitro evaluations using the MC3T3-E1 preosteoblast cell line showcased encouraging biocompatibility, cell attachment, and proliferation results. Displaying a porosity between 50% and 66% and efficient degradation, these scaffolds present potential for craniofacial defect repair [109]. However, some research reports challenges with metal ion methods, such as burst release, cytotoxicity, stability, and a short half-life. To mitigate these challenges, controlled-release coatings that harness synergistic effects are recommended. A rising trend points to smart release coatings, although many currently fall short in promoting bone growth. A future research focus is on dual-action coatings that can release antibacterial agents in slightly acidic settings and osteogenic factors in mildly alkaline conditions, optimizing bone formation [105].

#### 2.5.3. Tissue Engineering

Tissue engineering stands out as a notable field, offering potential bone substitutes that can function as bioactive materials to stimulate bone healing and growth. Recombinant bone morphogenetic proteins (BMPs), a subset of the transforming growth factor-beta (TGF-β) superfamily, offer promise in bone regeneration due to their ability to induce the differentiation of mesenchymal stem cells into bone-forming cells (osteoblasts) [110]. This property makes them valuable in various medical applications, including orbital floor repair. A significant breakthrough in this area came with the development and commercialization of recombinant human BMP-2 and BMP-7 (also known as osteogenic protein-1 or OP-1) [111,112]. Coupling these BMPs with biomaterials like demineralized bone and bioresorbable synthetic polymers, and incorporating growth factors like TGF-β, fibroblasts, and vascular endothelial growth factors (FGF, PDGF, VEGF), has been shown to enhance cell attachment and biocompatibility. For instance, Asamura and collaborators employed a gelatin hydrogel for the controlled release of BMP-2 over an extended period. The gelatin-BMP-2 system, coupled with a biodegradable copolymer, was evaluated in a canine orbital floor fracture model. Radiolabeled BMP-2 in gelatin hydrogel released slowly (approximately 60% at 3 days, 80% at 14 days), outperforming fluid-injected BMP-2 (over 90% lost in 8 h). The BMP-2-saturated gelatin hydrogel with a biodegradable copolymer promoted significant new bone formation and defect healing in 5 weeks, unlike direct copolymer saturation. Trabecular bone structure resembling normal tissue was restored by slow-release constructs, highlighting slow-release BMP-2′s utility for bone defect healing [81].

There are several commercially available carriers for delivering osteoinductive growth factors such as OP-1, INFUSE^®^, InductOS^®^, and AUGMENT^®^ [110]. OP-1, containing recombinant human BMP-7, type I bovine collagen matrix, and carboxymethyl cellulose sodium, was the first FDA-approved osteoinductive biomaterial. Multiple RCTs applying this material to orthopedic fractures have shown it to be effective and safe [113,114]; however, this material has yet to be tested in the orbit. INFUSE^®^ bone graft, containing BMP-2 and absorbable collagen sponge (ACS) as a carrier, is widely used in bone fractures, spinal fusions, and oral maxillofacial surgery. Clinical trials have demonstrated its efficacy, although it is associated with higher complication rates [115]. Moreover, a recent systematic review encompassing 17 RCTs investigating polymer-based BMP-2 delivery systems for craniofacial bone defects found no supportive evidence for the effectiveness of recombinant human BMP-2/ACS and BMP/hydrogels with HA in maxillary sinus augmentation and cranial vault defect reconstruction. Thus, further research is necessary to assess the efficacy of recombinant BMP-2/ACS and recombinant BMP/hydrogels for orbital repair [112].

In line with the functions of BMPs, recent investigations have highlighted the substantial role of platelet growth factors, including platelet-derived growth factor (PDGF) and transforming growth factor-β1 and β2 (TGF-β), in bone regeneration, particularly within the maxillofacial context. PDGF has been found to promote cellular proliferation, angiogenesis, and macrophage activation, while TGF-β enhances extracellular matrix deposition, chemotaxis, and mitogenesis among osteoblast precursors. Exploring these factors, polymer-based bone substitutes have been combined with allogeneic human platelet/cryoprecipitate mixtures, displaying encouraging outcomes for bone regeneration. Biphasic calcium phosphate ceramics, encompassing HA and β-tricalcium phosphate (β-TCP), are known promoters of new bone generation by releasing essential calcium and phosphate ions. Chen and colleagues investigated the potential of combining HA/β-TCP with platelet fibrin glue enriched with growth factors in a study involving 10 patients requiring orbital fracture repair. Their findings indicated that this approach facilitated simple application, molding, and secure fixation, effectively promoting the reconstruction of bone defects in a cost-effective manner. Although certain challenges were encountered, such as the requirement for allogeneic blood products, their conclusion pointed to the viability of this strategy as an alternative to conventional methods like autologous bone grafting, titanium mesh usage, or the application of recombinant BMP-2 in orbital floor reconstruction [116].

The combination of bone-marrow-derived mesenchymal stem cells (BMSCs) with biomaterials has demonstrated the ability to establish an osteoconductive environment, resulting in improved tissue regeneration and accelerated healing. In a study by Wang and colleagues, BMSCs in conjunction with β-TCP were explored for repairing canine orbital defects. Notably, the BMSC-BM/β-TCP group exhibited notable cell concentration within the graft, with sequential CT scans illustrating the gradual absorption of scaffolds and subsequent restoration of the defect. Detailed micro-CT and histological examinations confirmed the efficacy of defect repair within the experimental group, surpassing the outcomes observed in the control groups [117]. In another investigation, Deng and his team examined miR-31 genetically modified BMSCs combined with porous β-TCP scaffolds for repairing canine medial orbital wall defects (approximately 10 mm in diameter). CT scans revealed significant regenerative progress, specifically at the 16-week juncture post-surgery and within the genetically modified anti-MiR-31 group. Furthermore, micro-CT analysis indicated a discernible increase in bone mineral density and new bone volume in the anti-miR group. In contrast, the miR-31 group displayed reduced values of these parameters compared to the miR-negative group. Histological evaluation further validated the heightened formation of new bone and the extent of β-TCP scaffold degradation within the anti-miR group, while these aspects were suboptimal in the miR-31 group. The consistency of these micro-CT findings was further corroborated through in situ hybridization and immunohistochemical analysis [118].

#### 2.5.4. Patient-Specific Implants (PSI) and 3D Printing

In recent years, patient-specific implants (PSI) have made notable strides, allowing for tailored solutions to tissue loss challenges. For instance, Guillaume et al. developed a unique implant leveraging stereolithography (SLA). They crafted PSIs featuring laminated structures of poly(trimethylene carbonate) (PTMC) combined with biphasic calcium phosphate particles and supplemented with titanium mesh. These Osteo-PTMC implants, when compared to standard titanium mesh, fostered rapid neovascularization and bone growth in the orbital area without relying on additional biotherapeutics. Ultimately, they determined that the Osteo-PTMC composite had a distinct edge over the conventional titanium mesh [119]. Additionally, the emergence of 3D printing has significantly transformed the customizability of orbital biomaterials. An investigation by Sigron and colleagues compared traditional reconstructions, where 12 patients had their orbital mesh plate bent during the surgery, to an approach where 10 patients received plates pre-molded through 3D printing, tailored to reflect the patient’s intact orbit. Results showed that the 3D-printed approach led to a lesser volume discrepancy in the treated group and a more noticeable discrepancy in the conventional group. Moreover, surgeries using 3D-printed plates were markedly shorter, registering at 57.3 ± 23.4 min against 99.8 ± 28.9 min for the traditional method [99]. This underscores the precision and efficiency gains realized through 3D-printed reconstruction models [46]. Similarly, Blumer and colleagues evaluated the use of 3D-printed biomodels to customize titanium mesh for orbital fracture reconstruction in 34 unilateral orbital fracture cases. By mirroring the unaffected side, surgical precision was enhanced, resulting in fewer post-operative complications and reduced need for revisions. The approach significantly minimized long-term issues like diplopia and mildly sunken eyes [120]. Recently, Kwon and Shin compared the orbital reconstruction outcomes of 90 patients using 3D-printed customized implants versus conventional manual-bending implants. Results showed no significant difference in post-operative volume discrepancies between the two methods. However, larger fractures benefited more from the 3D-printed template approach, suggesting superior surgical outcomes with this method [121].

**Table 2:** Emerging Biomaterials for Orbital Floor Repair.

Material Type	Key Features	Advantages	Challenges	References
Additives and coatings	-Surface modifications of metals, ceramics and polymers -Include surface-treated titanium and porous coatings	-Some coatings offer antibacterial properties and promote osteointegration	-Potential cytotoxicity -Challenges with stability and long-term performance	[105]
Nanoparticles	-Incorporation of polyurethane (PU), HA, and antimicrobial metal ions into materials	-HA nanoparticles into cyclic acetal hydrogels have shown positive in vivo bone growth -HA synthesized with ionic substitutions can enhance bioactivity and cell viability -Antimicrobial ions enhance antimicrobial properties	-Potential cytotoxicity -Unclear stability -Short-half life	[105,106,107,109,110]
Tissue engineering	-Include the application of recombinant bone morphogenetic proteins (BMPs) and bone marrow-derived mesenchymal stem cells	-Shown to promote tissue regeneration and accelerated healing	-Limited research in human trials	[81,110,111,112,113,114,115,116,117,118]
Patient-Specific Implants (PSI) and 3D Printing	-Crafted from biomaterials such as poly(trimethylene carbonate) and titanium mesh	-Offer customizability and surgical precision -Shown to foster rapid neovascularization and bone growth	-Further research required to confirm long-term efficacy	[46,119,120,121]

### 2.6. Challenges and Barriers

In the realm of orbital floor repair, the task of selecting the most appropriate biomaterials is a dynamic challenge, constantly evolving in response to new discoveries, surgical practices, and patient needs. The diverse array of materials available today, while offering a variety of choices, also presents a spectrum of challenges that must be overcome to optimize patient outcomes. One primary challenge is the elusive pursuit of universality. In the past, different materials, when skillfully employed, have yielded satisfactory outcomes. Yet, no single material has emerged as a universally accepted or successful solution. Autologous implants, traditionally perceived as the gold standard, present their own set of challenges. Donor site morbidity and the heavy dependence on the surgeon’s experience make them a double-edged sword [16,122]. Allogeneic materials provide osteoinductive and/or osteoconductive properties without living cells, integrating into host tissue to serve as a scaffold for tissue growth. Despite advantages over autologous grafts, such as lack of donor site morbidity and reduced surgery time, they face challenges, including enophthalmos rates and higher resorption rates compared to autologous grafts, as well as the risk of disease transmission [19,20,21,22,23,24,25]. As we lean increasingly towards alloplastic materials, driven by their accessibility and ease of use, permanent materials pose foreign body risks while resorbable ones might not adequately support the orbit [26]. Resorbable materials can prevent complications like secondary displacement or graft extrusion, eliminating the necessity for removal [83]. However, resorption can trigger inflammation and their degradation may alter mechanical properties, potentially resulting in insufficient robustness to adequately support the contents of the globe in large fractures. Complications vary among materials, emphasizing the importance of careful selection based on factors like surgical technique, host response, and toxicity. Further research is needed to compare materials and develop guidelines for optimal selection in treating orbital fractures.

For ideal reconstruction, a biomaterial should be adaptable to the surgeon’s needs, capable of mechanical support, and able to facilitate native tissue regeneration without adverse biological reactions. For optimal reconstruction in orbital floor repair, the biomaterial selected must meet specific criteria tailored to the patient’s age and the nature of the injury. In adults, where communitive fractures necessitate the replacement of a substantial portion of the orbital floor, a permanent implant is often required to provide enduring support. Conversely, in pediatric cases characterized by ‘greenstick’ fractures with entrapped muscles, surgery aims to release the muscle, and a resorbable implant, such as PLA/PGA, can be used to prevent the recurrence of muscle entrapment at the fracture site. Therefore, while resorbability is a valued characteristic, it must be judiciously considered in the context of the fracture type and the patient’s age [16,122].

Future innovations should emphasize easy sterilization and adaptability for surgical needs, specifically focusing on ease of contouring and implantation. A significant limitation of available commercial biomaterials for orbital floor repair is their lack of bioactivity. The field of reconstructive surgery has seen remarkable advancements in orbital floor reconstruction through novel biomaterials that merge structural support with biocompatibility. Additive manufacturing, particularly in modifying materials like titanium, is increasingly prominent, with coatings such as those developed through microarc oxidation, dealloying, and 3D printing showing benefits for osseointegration [105]. Recent research has delved into nanoparticles, such as those made of PU and HA to enhance bioactive scaffolds for orbital repair, with studies revealing benefits like improved cell viability and mechanical strength [106,107,108]. Further, while incorporating ions into HA structures can boost bioactivity, the use of antibacterial metal ions in porous structures poses challenges, such as cytotoxicity and stability, necessitating advancements in controlled-release coatings [109]. In tissue engineering, BMPs have emerged as vital players, especially when combined with other biomaterials for sustained release, to promote bone growth. Commercially available carriers for these BMPs have shown effectiveness in orthopedics but require further trials in orbital applications [117]. There are also concerns about their efficacy in orbital repair and potential complications associated with commercially available carriers like INFUSE [115]. Studies integrating BMSCs with biomaterials also show potential for better tissue regeneration, but require further investigation before widespread clinical application [118].

Over the past decade, there has been significant advancement in PSIs, enabling precise implant fitting to address tissue loss. These implants are especially applicable for orbital floor and medial wall defects. Employing PSIs requires the accurate assessment of tissue loss through 3D virtual printing molds or prefabricated titanium implants. However, using such implants does not solely address the material’s suitability; it introduces the consideration of implant shape in addition to material choice. Further, there are limitations in addressing the inherent variation in distinct orbital morphologies, emphasizing the need for a more personalized approach to implant design and production [121]. To ascertain the superiority of a specific material for primary orbital floor fracture repair, there is a need for longitudinal comparative randomized studies, founded on a validated fracture classification [43]. Overall, while these advancements show immense promise, addressing research gaps, especially regarding the long-term efficacy and safety of these novel materials, will be critical in determining the future direction of orbital reconstruction techniques.

### 2.7. Gaps in Knowledge and Future Directions

The future of orbital floor reconstruction should ideally amalgamate the benefits of these innovative materials, considering both their material properties and biological effects. Collaboration among experts from various fields can pave the way for selecting and marketing the most suitable and economically viable biomaterials to enhance patient outcomes. As the menu of biomaterials expands, the onus is on the medical community to ensure their integration into surgical practice is underpinned by rigorous, wide-ranging research. Few studies exclusively compare reconstructive materials for orbital fractures, necessitating further research for standardized comparisons and guideline development. Concurrently, the existing body of research and practical experience has yet to converge into comprehensive, universally endorsed guidelines for material selection in orbital fracture treatments [123]. Peering into the future, the plethora of emerging biological and synthetic polymers accentuates the need for an intensive review of their long-term effects and their suitability for more widespread adoption. This foundational research should encompass both laboratory experimentation and clinical evaluations in real-world settings to thoroughly gauge their safety and effectiveness.

## 3. Role of Functional Biomaterials in Orbital Implants and Prothesis

### 3.1. Overview of Orbital Implants and Prosthesis

Orbital implants and prostheses play a crucial role in restoring facial symmetry and aesthetics in patients who have undergone surgeries such as evisceration, enucleation, or exenteration.

Evisceration involves the extraction of the globe’s contents while leaving the sclera, extraocular muscles, and optic nerve intact [124]. This procedure maintains the relationships between the muscles, globe, eyelids, and fornices undisturbed, offering better prosthesis motility and easier fitting [125,126,127]. However, it is strictly avoided if there is a suspicion of an intraocular malignancy.

Enucleation, the removal of the eye while preserving the orbital tissues, is primarily executed for intraocular malignancies like retinoblastoma and choroidal melanoma that are unresponsive to other therapies [124]. The procedure ensures complete histological examination, reducing the theoretical risk of disseminating tumor cells and the potential for sympathetic ophthalmia.

Exenteration entails the removal of the entire globe and some or all of the orbital soft tissues, reserved for extreme cases like destructive tumors extending into the orbit from surrounding tissues or primary orbital malignancies unresponsive to other treatments [128]. The extent of the exenteration varies, from subtotal, which involves the local excision of the lesion, to extended, which entails the removal of adjacent structures including bony walls and sinuses.

**Figure 2:**
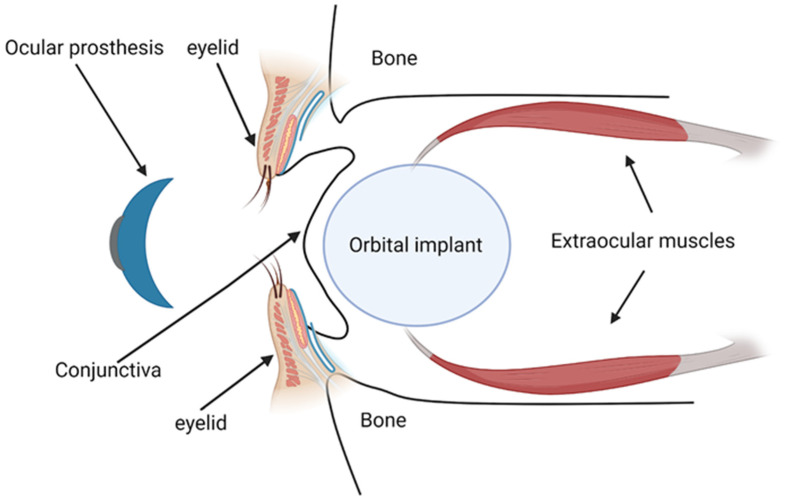
Anatomical diagram showing an orbital implant placement with an overlying ocular prosthesis, integrated with the extraocular muscles and eyelid structure.

Orbital implants are primarily designed to replace the lost orbital volume and maintain the structure of the orbit [129] (Figure 2). By imparting motility to the overlying ocular prosthesis, they also help restore eye movements, enhancing the natural appearance [129]. These implants may be categorized based on the materials used for their manufacturing. Inert materials, which include glass, silicone, or methylmethacrylate, constitute one category [7]. These materials provide comfort, have low extrusion rates, and are cost-effective, making them suitable for patients not requiring implant integration. However, their application may be limited due to the potential for implant migration [130].

On the other hand, biointegrated materials, such as hydroxyapatite, porous polyethylene, and aluminum oxide, are designed to be incorporated into the soft tissue from the socket [7]. These implants allow for the direct attachment of extraocular muscles, facilitating the direct integration of the prosthesis with the moving implant. This integration imparts excellent motility, enhancing the cosmetic outcome, although it may increase the risk of post-operative complications such as exposure and infection [7].

The prosthesis, typically custom-fitted four to twelve weeks post-operation, is designed to the exact dimensions of the patient’s conjunctival fornices [7] (Figure 2). It further enhances cosmetic results by providing the external appearance of the eye, matching the companion eye in color, size, and position.

### 3.2. Indications for the Use of Orbital Implants and Prosthesis

The use of orbital implants and prostheses is typically indicated in cases where surgery involving the removal of part or all of the ocular and orbital tissues is necessary. These surgical procedures are usually categorized into enucleation, evisceration, and exenteration.

These procedures necessitate the use of an orbital implant to maintain the structure and volume of the orbit, and a prosthesis for aesthetic purposes. The surgical and cosmetic goals of these procedures focus on minimizing any condition that draws attention to the anophthalmia [131]. Surgical efforts aim to maximize orbital implant volume, achieve optimal eyelid contour, establish a socket lining with deep fornices, transmit motility from the implant to the overlying prosthesis, and achieve comfort and symmetry [131]. The success of these procedures is marked by their ability to achieve these goals and restore facial symmetry.

### 3.3. Ideal Properties of Biomaterials for Orbital Implants and Prosthesis

Choosing the right orbital implants and prostheses materials plays a vital role in maximizing the success of anophthalmic surgeries and enhancing the quality of life for patients. An optimal selection hinges on a range of properties that ensure the integration, functionality, and aesthetic appeal of these devices.

Orbital implants should have the following ideal properties:

- Biocompatibility: The material should be non-allergenic, non-toxic, and not incite an adverse immune response from the host tissue.
- Long-term acceptance: The material should either be permanently accepted by the body.
- Manipulability and stability: The material should be easily manipulated during surgical procedures and retain its form post-implantation. It should maintain sufficient volume to maintain the natural structure of the orbit.
- Mechanical stability and motility: Implants should allow natural movement of the prosthesis for optimal aesthetic outcomes.
- Proper support for ocular prosthesis: Implants should hold and support the prosthesis appropriately.
- Cost-effectiveness: Implants should be economically accessible for a wide range of patients.
- An optimal ocular prosthesis should possess the following ideal attributes:
- Lightweight: To promote comfort, the prosthesis should not be heavy.
- Color match: The prosthesis should match the color of the contralateral eye for a natural appearance.
- Hygiene: The design should facilitate easy and effective cleaning.
- Texture: The prosthesis should mimic the natural eye to provide a realistic look and feel.
- Availability: The prosthesis should be easily accessible for replacements or adjustments as needed.

When considering porous orbital implants, biocompatibility, safety, and long-term acceptance become especially significant. Made from biointegrated materials, these implants are designed to fuse with the surrounding tissue, facilitating extraocular movements and enhancing the natural appearance [7]. The process entails the attachment of extraocular muscles to the implant. Complete vascular biointegration typically occurs 6–12 months after enucleation, enabling excellent motility [7]. However, this porous implant also introduces an elevated risk of post-operative complications, including exposure, extrusion, and subsequent infection [7]. Therefore, ensuring the implant’s biocompatibility, safety, and sustained acceptance by the body is crucial to minimize complications and optimize both functional and aesthetic outcomes with porous biointegrated implants.

### 3.4. Current Gold Standard, Alternative Options, and Types of Biomaterials in Orbital Implants and Prosthesis

An overview of the key features, advantages, and disadvantages of current biomaterials used for orbital prostheses is described below and summarized in Table 3.

#### 3.4.1. Ceramics

Mules introduced the first orbital implant in 1885, crafted from a hollow glass sphere [132]. This ceramic implant, due to its non-crystalline oxide-based nature, was the standard until the 1940s. However, its fragility has made its use obsolete, with rare exceptions when patients were unable to tolerate other materials. In subsequent decades, the simple designs of early non-porous polymeric spheres evolved into more intricate and functionally advanced designs to improve long-term clinical results. Notably, ceramic and polymeric porous implants gained traction because their interconnected porous designs permitted fibrovascular growth, improving the longevity of the implant and enabling better movement of the prosthetic eye [133].

HA, derived initially from bovine bone, has become an applied material in this arena. Specifically, in the 1990s, coral-derived HA gained popularity due to its similarity to the structure of human bone [134] with synthetic HA later emerging as a cheaper and equally effective alternative. Both versions of HA allow for vascular growth within their porous structures [134,135]. Yet, HA implants have notable limitations. HA’s porous ceramic nature complicates suturing, and its rough surface can impact biocompatibility, often requiring a wrapping material. While complications exist, like conjunctival thinning and infections, treatments to resolve these are available, and removal might be necessary in severe cases. The use of HA implants in children is also debated due to growth-related concerns [136,137].

Aluminum oxide (Al_2_O_3_), often referred to as alumina, has been integral in orthopedics and dentistry due to its mechanical properties, biocompatibility, and bioinertness. The material also encourages fibroblast growth and integrates with orbital tissue, owing to a protein coating that acts as an “immune camouflage”. Initial animal studies showcased the potential of porous alumina, with evidence of rapid fibrovascular in-growth and minimal complications [138,139]. Alumina has been found to be comparable in biocompatibility to HA but with the added advantage of being more cost-effective. Notably, a study of this material in 107 patients reported rare post-operative complications, with an implant exposure rate of only 9.1% [140]. However, recently Cameron and colleagues reported a case wherein a porous alumina implant wrapped in polyglactin 910 led to extensive orbital inflammation [141]. Research by Ramey and collaborators also found higher complication rates with alumina and porous PE than with HA implants [140,142,143]. To optimize the outcomes associated with porous alumina, some researchers have suggested combining these materials with autologous tissues. For instance, Zigiotti and colleagues introduced a procedure that involved partially covering the implant with the patient’s own sclera, resulting in no implant extrusions and satisfactory prosthetic outcomes after 16 months [144]. Wang and Lai also successfully utilized a retroauricular myoperiosteal graft alongside alumina, ensuring the implant’s coverage and avoiding recurrence for over 2 years [145].

#### 3.4.2. Autologous Materials

Orbital implants typically utilize synthetic materials such as polymers and ceramics. Yet, in certain instances, autologous tissues may be recommended for restoring orbital volume. Economic considerations often influence this choice in adults, as synthetic implants come at a higher cost. For children, whose tissues and anatomical structures evolve over time, autologous tissues are sometimes preferred [146]. Dermal fat grafts, known for their growth potential, are suggested for both primary and secondary enucleation [147,148,149]. However, their success rate can be uncertain, hinging on the blood supply of the receiving tissue which might be impaired post-surgery or after radiotherapy for ocular tumors. Alternatives encompass cortical and cancellous bone grafts combined with temporalis muscle flaps [150], anterolateral thigh flaps [151], and post-auricular skin grafts [152]. In situations where patients cannot tolerate synthetic materials in the anophthalmic socket, manifesting symptoms like persistent inflammation or chronic pain, a dermis fat graft implantation is often recommended [153]. Beyond serving as orbital implants, autologous tissues are frequently used to wrap or salvage exposed implants [154]. Some studies have also documented the combination of a smaller porous orbital implant with a periumbilical fat autograft to augment socket volume after enucleation, aiming to minimize the risk of implant exposure [155]. Using autologous materials comes with its own set of challenges, including the necessity of an extra surgical procedure and potential post-operative issues at the tissue extraction site.

#### 3.4.3. Polymers

##### Silicone

As described earlier, silicone has been favored in various surgical procedures due to its inertness, flexibility, affordability, and ease of use. While typically well-tolerated, occasional complications such as inflammation, increased intraocular pressure, and implant migration have been noted. Over the years, a myriad of silicone implant designs have been crafted to address varied surgical needs [156]. Introduced by Girard and McPherson, solid silicone implants have been used to resolve scleral erosion issues observed with earlier implants. However, their restricted impact on ocular circumference gave rise to flat silicone bands for enhanced lateral support [157]. The bands’ widths have evolved, and to secure them, non-magnetic, biocompatible tantalum clips were devised, along with round silicone sleeves. Additionally, grooved silicone strips, silicone “tyres”, and meridional implants have been developed to offer diverse buckling configurations and mitigate post-operative retinal puckering [158].

In the 1960s, sponge silicone implants presented a flexible counterpart to solid ones, obviating the need for external drainage in many retinal detachment surgeries. Available in designs, such as radial placements for small breaks and circumferential buckling for larger tears, their thickness has been reduced over time to prevent complications. Other innovations include hollow and L-shaped sponges for specific use cases. Efforts to enhance sponges with antibiotics were deemed largely superfluous [156]. In a study by Son and colleagues, experimental porous silicone orbital implants were compared to commercial Medpor in New Zealand white rabbits. Four weeks post-surgery, porous PE implants exhibited more fibrovascular in-growth than porous silicone (42.4% vs. 34.2% of implant radius), with a similar pattern seen after 8 weeks (71.6% vs. 63.6%). These findings suggest porous silicone might offer an affordable alternative to current implants, but further extensive research is essential to confirm its efficacy and benefits.

##### Poly(methylmethacrylate) (PMMA)

Poly(methylmethacrylate) is a prevalent polymer for ophthalmic use due to its biocompatibility and clear visibility, especially in intraocular lenses, rigid contact lenses, orbital implants, and orbital prostheses. It comes in non-porous and hollow variations, with the latter proposed to reduce weight and risk of ectropion [159]. An earlier study by Groth determined that PMMA orbital implants did not lead to major complications, and patients benefited from its tailored design, lasting biocompatibility, and superior aesthetic results, over an average follow-up period of 4.3 years [160]. Several years later, Taneja and colleagues retrospectively assessed 321 retinoblastoma patients who underwent unilateral enucleation with a primary orbital PMMA implant. Over an average follow-up of 40 months, 9% experienced implant migration, 3% had implant extrusion, 2% saw implant exposure, and 2% had socket contraction. Only four (1%) required implant exchanges for a better prosthesis fit. They concluded that a PMMA implant post-enucleation for retinoblastoma offers satisfactory cosmetic results with few complications [161]. Recently, Silva and colleagues evaluated the clinical and tissue response of a hollow PMMA orbital implant with a multiperforated posterior surface in a rabbit model after evisceration. There were no signs of infection, conjunctival or scleral thinning, or implant exposure or extrusion in any animal during the 180-day study period. On day 7, the new tissue migrated into the implant and formed a fibrovascular network through the posterior channels. Inflammatory response reduced over time, and no multinucleated giant cells were found at any time, concluding its safety for human trials [133].

In terms of ocular prostheses, off-the-shelf and custom-made PMMA devices dominate the current market, with PMMA being preferred due to its enhanced durability, longevity, and tissue compatibility. However, PMMA is relatively hydrophobic, which can increase the likelihood of deposits of tear proteins and other debris settling on the prosthesis’s front surface between the eyelids hindering the formation of a stable tear film. This can lead to poor wettability, patient discomfort, and anophthalmic socket conditions, such as dryness, lacrimal drainage blockage, meibomian gland dysfunction, excessive mucoid discharge, and lagophthalmos. To resolve this, Pine and colleagues explored different derivatives of poly(ethylene glycol) (PEG) monomer and methyl methacryte (MMA) monomer grafted to PMMA to improve PMMA’s hydrophilic properties. In their study, they determined that ethylene glycol dimethylcrylate (EGDMA) grafted PMMA exhibited the best hydrophilic performance than the other biomaterials tested. This was shown to reduce contact angles significantly, improving wettability by 33% compared to polished PMMA prosthetic eyes. Thus, EGDMA-grafted PMMA may offer a practical solution for PMMA’s hydrophobicity, surpassing other prosthetic solutions [162].

##### Poly-HEMA (2-Hydroxyethyl Methacralate) Implant (Alphasphere)

In 2006, the FDA approved Alphasphere, an orbital implant made of poly-HEMA (2-hydroxyethyl methacrylate). This implant features a unique two-phase structure, with a porous sponge at the front and a non-porous gel at the back. This innovative design combines the benefits of porous implants while leveraging the non-porous posterior section to prevent tissue integration, thereby enhancing mobility. A study conducted by Shevchenko and colleagues, involving 12 recipients of Alphasphere implants, demonstrated the implant’s effectiveness in managing anophthalmic sockets. It offered various advantages, such as eliminating the need for tissue wrapping, facilitating direct muscle suturing, reducing exposure risks, and optimizing the placement and maneuverability of prostheses. Notably, only one patient required implant removal due to a sinus infection, while another experienced slight implant shifting [163]. However, Yadav and his team presented a case of a 54-year-old diabetic patient who underwent a secondary Alphasphere implant after enucleation due to endophthalmitis. Six weeks later, the implant’s deterioration necessitated removal, with further investigation revealing it prompted a severe and irreversible host tissue reaction without signs of infection [164]. In a case highlighted by Neimkin et al., a 57-year-old female who underwent enucleation for choroidal melanoma and received an Alphasphere implant faced implant failure after 2 years, despite an initially uneventful recovery. The observed issues prompted an implant exchange and underscored the need for more research on this implant’s long-term efficacy.

##### Polytetrafluoroethylene (ePTFE or Gore-Tex) Based Materials

Other attempts at polymeric orbital implants include Gore-Tex spheroids, which were found unsuitable due to inflammation. In the 1990s, Dei Cas and colleagues explored the potential of expanded ePTFE spheres as orbital implants using six New Zealand white rabbits. Post-enucleation, the rabbits received spherical Gore-Tex implants. Six weeks later, none showed post-operative infections, exposure, or extrusion, but there was inflammation and fibrovascular growth into the implants up to 500 μm deep. The inflammation observed around each implant might explain the lack of further studies on Gore-Tex for orbital implants in subsequent years [165]. Similar inflammatory issues with ePTFE were noted by Mortemousque and colleagues when used for retinal detachment treatments, possibly due to its mismatched stiffness with orbital tissues [166].

In the late 1970s, Lyall innovatively used Proplast, a blend of ePTFE and carbon fibers, to create hemispherical orbital implants. These implants were designed for fibrous tissue integration, reducing risks of extrusion and rejection. After an 18-month follow-up, the 16 patients with these implants reported no rejections and had satisfactory motility [167]. Neuhaus and colleagues also found promising results with Proplast implants in rabbits and humans, noting high tissue fixation and no migration [168]. Whear and team conducted a 9-year retrospective study on 36 cases with 88 Proplast implants, identifying infections as the predominant post-operative complication, with an elevated infection rate of 16% compared to previous studies [169]. This heightened risk is one of the primary reasons behind Proplast’s declining usage in recent years.

Girard and team introduced a unique porous, quasi-integrated enucleation implant named Proplast II, which combined the benefits of fibrovascular in-growth and enhanced motility. Unlike its predecessor, Proplast I, Proplast II was made of ePTFE and alumina, with a design that featured a smooth, non-porous back for better movement and a porous front for tissue integration [170]. Although some Proplast II implants were removed due to motility issues, they have been utilized as subperiosteal implants to address anophthalmic enophthalmos in patients with inadequate orbital volume replacement [171,172].

**Table 3:** Current Biomaterials for Orbital Implants and Prosthesis.

Material Type	Key Features	Advantages	Challenges	References
Ceramics	-Initially derived from glass -HA and alumina more commonly applied	-Porous designs permit fibrovascular growth that enhances implant longevity -Good outcomes when combined with autologous tissues	-Porous ceramic structure complicates suturing -Rough surfaces can impact biocompatibility -Limited application in children due to growth-related concerns	[132,133,134,135,136,137,138,139,140,141,142,143,144,145]
Autologous materials	-Include fat, bone, skin, cartilage and muscle grafts	-More affordable than synthetic implants -Useful for patients who cannot tolerate synthetic materials or children whose anatomical structures evolve over time -Used to wrap or salvage exposed implants	-Unpredictable success rate -Potential post-operative complications at tissue extraction sites	[146,147,148,149,150,151,152,153,154,155]
Silicone	-Various designs including solid, grooved strips and sponge-based implants -Porous and non-porous forms	-Biologically inert, reducing risk of adverse reactions -Flexibility and ease of handling	-Limited prosthetic movement compared to other implant types -Risk of migration without proper wrapping or connections	[156,157,158]
Poly(methylmethacrylate) (PMMA)	-Non-porous and hallow variations	-Highly biocompatible and transparent to visible light -Provide good volume correction -Offer stability and customizability -Positive aesthetic results	-Hydrophobicity can increase deposits of tear proteins and other debris near eyelids -Risk of poor wettability leading to dryness, lacrimal drainage blockage, meibomian gland dysfunction, excessive mucoid discharge, and lagophthalmos	[133,159,160,161,162]
Poly-HEMA (2-hydroxyethyl methacralate) implant (Alphasphere)	-Two-phase structure with a porous sponge at the front and non-porous gel at the back	-Does not require tissue wrapping -Facilitates direct muscle suturing -Enhanced mobility	-Reports of host tissue reactions and implant failure	[163,164,173]
Polytetrafluoroethylene (ePTFE or Gore-Tex) based materials	-Porous quasi-integrated enucleation implant -Siliconized non-porous posterior surface -Composites of Teflon/alumina and Teflon/carbon fibres	-Composite materials have shown reduced complication risks -Enhanced motility	-Inflammatory complications limit application of pure Teflon implants -Risk of infection	[165,166,167,168,169,170,171,172]
Porous PE (Medpor)	-Made from ultra-high molecular weight polyetheylene	-Minimal inflammation and fibrosis -Cost-effective -Smoother surface -Affordable	-Slower vascularization than other implant materials -Poor tissue in-growth can limit antibiotic penetration, necessitating implant removal in cases of infection -Advanced designs with added functionalities are more costly	[174,175]

##### Polyethylene (PE)

As described earlier, synthetic porous PE implants, such as Medpor^®^, have gained traction in oculoplastic applications. They present advantages over HA by causing less inflammation and offering a smoother surface. However, they may exhibit slower vascularization, dependent on pore size. PE’s appeal lies in its cost-effectiveness, versatility in design, and ability to bond directly to extraocular muscles, eliminating the need for a protective scleral wrap. Notably, while Medpor can function with or without wrapping, some studies, like one by Jung and colleagues, have linked HA wrapping with increased exposure rates. In their 50-month follow-up study involving 314 patients with Medpor orbital implants, the primary complications noted were blepharoptosis (10.5%), exposure (4.5%), and implant infection (1%). Thankfully, all these complications were remedied either surgically or through conservative care. The study deduced that Medpor’s surgical results were similar to other porous orbital implants in terms of the nature and frequency of complications [174]. Lin and Liao evaluated the long-term results of various orbital implants and the effectiveness of dermis fat grafts for significant implant exposure. Exposure rates exceeded previous reports, with the highest rates in the Medpor category (76.5%) in comparison to HA (24.7%) or bioceramic (23.5%) materials. Late exposures in porous orbital implants, particularly in eviscerated globes and pegged implants, were common, with dermis fat grafting showing efficacy in managing extensive exposures [175].

### 3.5. What’s New? Emerging Biomaterials and Their New Applications in Orbital Implants and Prosthesis

An overview of the key features, advantages, and disadvantages of emerging biomaterials used for orbital implants and prostheses is described below and summarized in Table 4.

#### 3.5.1. Surface Coatings

A novel method currently being researched to improve the efficacy of orbital implants involves the application of unique surface coatings. These coatings are designed to either stimulate fibrovascularization or provide antibacterial properties, elevating the implants’ performance beyond the current standards.

##### Coatings to Improve Vascularization

In the early 2000s, You and colleagues initially aimed to boost vascularization by adding a synthetic HA coating to alumina implants, capitalizing on alumina’s load-bearing properties and HA’s biocompatibility [176]. Their tests on eviscerated rabbits showed fibrovascularization at the implant’s edges within 2 weeks and its center by 4 weeks. Seong’s team also studied these implants in rabbits, noting fibrovascularization, especially in those with 500 μm pores [177]. However, a subsequent study by Jordan and colleagues on calcium phosphate coatings found no significant impact on fibrovascular growth in rabbits, leading to the discontinuation of this approach [178]. Later, Chung and collaborators also found no significant difference between HA-coated porous alumina implants and HA spheres, except for a brief period post-operation [179]. Few studies have further investigated HA-coated implants, perhaps due to the lack of a clear clinical advantage and some manufacturing concerns.

Cui and colleagues demonstrated that, among 41 patients who received both basic fibroblast growth factor (bFGF) and antibiotic drops compared to those treated with only antibiotic drops, the bFGF group experienced notably improved healing rates, especially in cases of mild exposures [180]. In a different study involving 30 New Zealand rabbits, porous PE orbital implants were treated with bFGF either through soaking or injection. This resulted in a significant increase in fibrovascular ingrowth. Interestingly, the method of bFGF application did not influence the result, pointing to its potential to prevent complications, such as implant exposure [181]. In 2014, Liu and colleagues also examined the role of bFGF in promoting fibrovascularization in orbital implants using enucleated New Zealand rabbits. They compared three types of implants: naked HA, HA combined with a collagen sponge, and HA paired with a bFGF-treated collagen sponge. Histological evaluations conducted over 12 weeks post-surgery showed that implants with the bFGF-treated sponge had accelerated and thorough vascularization. In contrast, the collagen sponge by itself only aided early-stage fibrovascularization, proving less effective than the conventional HA implant [182,183].

In 2016, Jin and collaborators explored the use of porous HA scaffolds coated with VEGF functionalized collagen (COL)/heparin (HEP) using a layer-by-layer assembly (porosity 75%, pore size 316.8  ±  77.1 μm, VEGF dose 3.39 ng/mm^3^) to boost fibrovascularization through the conversion of mesenchymal stem cells (MSCs) into endothelial cells. They demonstrated that these specially coated scaffolds had enhanced mechanical strength, increased human cell proliferation in vitro, and displayed more pronounced angiogenesis compared to uncoated HA scaffolds [184]. Notably, in vivo studies confirmed more new vessels formed in the HA/(COL/HEP)5/VEGF/MSCs group than in other tested groups, with both mRNA and protein levels of specific markers also being highest in this group [185].

##### Coatings to Improve Antimicrobial Activity

In ophthalmology, preventing bacterial colonization on ocular prostheses is crucial, as infections often necessitate additional surgeries, posing added risks and stress to patients. Ions and nanoparticles, especially those of silver and copper, incorporated into orbital implants can disrupt bacterial cell functions and inhibit biofilm formation, offering a potent antimicrobial defense and reducing reliance on antibiotics. Yang and colleagues demonstrated that artificial eyes made with PMMA resin infused with 300–700 ppm silver exhibited potent antibacterial effects, reducing bacterial growth by 99.9% [186]. In a later study, Baino and his team applied a robust silver nanocluster/silica composite to a PMMA prosthesis, which exhibited strong adhesion and potent antibacterial action against Staphylococcus aureus. This method, leveraging metal ions over antibiotics, not only sidesteps bacterial resistance concerns but also alleviates potential toxicity by releasing silver as ions instead of nanoparticles [187]. Ye and collaborators introduced a CuO-doped mesoporous bioactive glass (Cu-MBG) coating over a porous HA orbital implant. This design utilized the vast surface area of mesoporous bioactive glass to amplify the implant’s drug-loading/releasing capacity, allowing it to discharge antibacterial copper ions as it decomposes. In vitro tests validated this two-fold anti-inflammatory and antimicrobial effect [188].

#### 3.5.2. Bioactive Glasses (BGs)

Bioactive glasses (BGs) are now being considered for ocular implants in oculo-orbital surgery due to their biocompatibility, cellular regeneration, and self-repair abilities. These glasses can release therapeutic ions for antibacterial or anti-inflammatory effects when integrated with specific elements. BGs are also valuable for inducing angiogenesis and fibrovascularization without promoting bone cell activity, which helps prevent bacterial colonization and treat infections [189]. An early in vivo study by Xu and colleagues involving glass-ceramic porous spheres in enucleated rabbits demonstrated vascularization of up to 90% of the porous volume over 6 months, with a faster fibrovascular response compared to porous PE [190]. These spheres were later used in 102 patients, yielding satisfactory results in terms of cosmetic appearance and ocular mobility over 6 to 24 months. Only minor complications, mainly due to surgical techniques, were reported [191]. Another noteworthy application by Heringer and Ng involved filling old pegged tracts in hydroxyapatite porous orbital implants with bioactive glass to correct peg mispositioning, which yielded successful outcomes over a 3-year follow-up period [192]. Wang and team used rabbit models to demonstrate that Cu-enhanced bioactive glass was proven to significantly boost early-stage vascularization within the scaffold’s porous architecture in comparison to Cu-free materials [193].

Drawing inspiration from earlier research, Porex Surgical (Newman, GA, USA) investigated the potential of incorporating bioactive glass particles into Medpor. By blending melt-derived 45S5 Bioglass^®^ particles into Medpor, they aimed to enhance fibrovascularization rates. Consequently, the resultant glass/PE composite, trademarked as Medpor^®^ Plus™ Sphere, received FDA clearance in 2002 and has since been globally available. However, initial research by Choi and colleagues on rabbits indicated that the inclusion of bioactive glass did not significantly impact fibrovascular ingrowth compared to glass-free Medpor [194]. Contrarily, a clinical trial by Naik and team on 10 human patients revealed improved fibrovascularization rates in those with glass-containing implants [195]. Another study by Ma and collaborators, examining 170 human patients with Medpor^®^ Plus™ Spheres, showcased mostly positive outcomes. However, without a direct comparison to glass-free Medpor^®^, it remains unclear whether the composite spheres provide a definitive clinical advantage [196].

#### 3.5.3. Biosilicate-Derived Implants

In 2010, Biosilicate^®^ (composition 23.75Na_2_O–23.75CaO–48.5SiO_2_–4P_2_O_5_ wt%) was introduced as a novel biomaterial for glass-ceramic orbital implants designed to restore volume in the anophthalmic socket. Originally conceptualized as an alternative to 45S5 Bioglass^®^ for bone and dental applications, this material also showed effectiveness when in contact with soft tissues, a crucial feature for orbital implants [197]. Notable studies by Brandão and colleagues and subsequent research demonstrated its biocompatibility and reduced inflammatory response in eviscerated rabbits [198,199]. This promising outcome led to a clinical trial between 2013 and 2016, where Biosilicate^®^ 1P implants were tested on 45 patients and yielded positive results. In particular, Biosilicate^®^ tapered implants, despite not having a macropore network, showcased impressive biocompatibility and bactericidal activity, enhancing their potential for clinical application. This groundbreaking work highlights the need for continued exploration into the biological responses elicited by such materials in future studies [197].

**Table 4:** Emerging Biomaterials for Orbital Implants and Prosthesis.

Material Type	Key Features	Advantages	Challenges	References
Coatings to improve vascularization	-Include synthetic HA, fibroblast growth factor (bFGF), and VEGF functionalized collagen	-bFGF coated with antibiotic drops has demonstrated improved healing rates and increased fibrovascular growth -Porous HA scaffolds coated with VEGF functionalized collagen may have enhanced mechanical strength, increased cell proliferation, and pronounced angiogenesis compared to uncoated HA scaffolds	-Lack of benefit of HA-coated implants -Current research only involves animal and in vitro studies	[176,177,178,179,180,181,182,183,184,185]
Coatings to improve antimicrobial activity	-Include silver, silver/silica, and copper-oxide nanoparticles	-Antimicrobial benefits	-Risk of toxicity	[186,187,188]
Bioactive glasses	-Porous and non-porous forms that can release therapeutic ions	-Biocompatible -Incorporation of ions enables antibacterial and anti-inflammatory effects -Induce angiogenesis and fibrovascularization	-Lack of long-term clinical evidence	[189,190,192,193,194,195]
Biosilicate-derived implants	-Enhanced glass composite with Na-Ca silicate	-Highly biocompatible -Reduced inflammatory response	-Novel application in ocular prostheses with limited evidence to date	[197,199]

### 3.6. Challenges, Barriers, Gaps in Knowledge and Future Directions

In summary, the journey of orbital implants from their rudimentary glass beginnings to the advanced biomaterials of today underscores the continuous pursuit of an optimal balance between function, appearance, and biocompatibility. As researchers continue to refine these devices, patients stand to benefit from even safer and more effective eye replacement options. The materials chosen for orbital implants are critical, given their lifelong function in maintaining socket volume and supporting orbital tissues. While substances like bioresorbable polymers and phosphate glasses might be apt for other roles, their potential for in vivo degradation makes them less suitable for orbital implants. Bioactive glasses, recently favored in ocular biomaterial research, demand precise composition to ensure their desired physicochemical and biological characteristics. They might offer a more economical option compared to other bioceramics. In scenarios where traditional procedures don’t suffice, dermal replacements could be a viable alternative, even if their use around the eyes is limited; available data, though scarce, suggests promising results.

Emerging biomaterials for orbital implants and prostheses encompass surface coatings, bioactive glasses, and Biosilicate^®^. Surface coatings, like synthetic HA, have demonstrated potential in stimulating fibrovascularization in orbital implants, with some studies reporting success in eviscerated rabbits [176]. However, the clinical advantage of HA-coated implants in human subjects remains unclear as some studies have shown no advantage to this approach [179]. Incorporating bFGF has shown improved healing rates, particularly in cases of mild exposures [180]. On the antimicrobial front, silver [186] and copper [188] nanoparticles incorporated into orbital implants have effectively inhibited bacterial growth, offering a promising antimicrobial defense. Bioactive glasses are gaining attention for their biocompatibility, cellular regeneration, and self-repair abilities [189], but conflicting clinical study results question their definitive advantage [196]. Biosilicate^®^ has shown biocompatibility and reduced inflammatory responses in animal studies [198,199] and yielded positive clinical trial results, marking a notable breakthrough [197]. Nevertheless, continued exploration is essential to understand biological responses and potential long-term complications associated with these emerging materials for orbital implants.

### 3.7. Gaps in Knowledge and Future Directions

It is imperative to ensure new implants meet their intended purpose. Currently, there is no standard method for their assessment. Based on the existing literature and expert opinions, initial solubility and in vitro compatibility tests are recommended. For bioactive variants, tracking ion release is crucial, excluding any potentially harmful materials to ocular tissues. To make the orbital implant selection more objective, introducing a measurable “selection score” might be beneficial, reducing dependence on individual surgical judgment. Using a benchmark from tissue engineering, a quantitative standard could aid in determining how well a scaffold mimics the tissue it replaces. In the patent landscape, while many orbital implants were developed years ago, recent times have seen fewer innovations. These new patents could link research to real-world applications [129]. However, many have not been put to clinical use or featured in academic publications. A noticeable gap exists between technological advances and their practical implementation in clinical settings. Lastly, there is a clear need for more comprehensive treatment guidelines related to post-operative management with prosthetic eyes. Further studies are necessary to better understand the socket’s reaction to prosthetic eyes, considering factors like the conjunctiva, socket fluids, and deposits on the prosthetic surface.

## 4. Limitations

This review summarizing biomaterials for orbital floor repair and orbital implants and prostheses acknowledges several limitations that warrant consideration. Firstly, the diverse range of biomaterials discussed in the literature poses challenges in making direct comparisons and drawing conclusive insights. Variability in study methodologies, sample sizes, and follow-up periods across different investigations further contributes to the complexity of synthesizing findings. Additionally, the evolving nature of the field results in a limited number of long-term clinical studies, hindering a comprehensive understanding of the durability and potential complications associated with these emerging materials. Many studies of newer biomaterials focus on animal models, and while they provide valuable insights, the translational relevance to human applications remains a subject of ongoing investigation. The lack of standardized assessment criteria for success, complications, and functional outcomes across studies impedes the establishment of a universally applicable benchmark for the efficacy of these biomaterials. Furthermore, this review recognizes the need for continued research to address gaps in knowledge, particularly regarding the long-term biocompatibility, immunogenicity, and potential adverse effects of materials. Finally, rapid technological advancements, while promising, highlight the importance of continually updating the latest developments in the field to refine our evolving understanding of emerging biomaterials.

## 5. Conclusions

In summarizing this review on functional biomaterials in orbital surgery, we recognize the significant advancements made in recent years. This synthesis of the literature reveals the extensive application of these materials in both repairing orbital floor injuries and creating orbital implants and prostheses post-eye removal. Our analysis of their pathophysiology, treatment indications, and optimal properties paves the way for future research and clinical advancements.

Notably, this review merges clinical insights with material science, offering a comprehensive view that spans from research to patient care. It addresses the challenges of adopting new materials and technologies in clinical settings and suggests ways to overcome these barriers. Emphasizing an interdisciplinary and patient-centered approach, this review highlights the crucial role of collaboration between material scientists and clinicians in enhancing patient outcomes in orbital surgery and related fields.

## Data Availability

Not applicable.
